# Life-Saving Emergency Decompression via a Clamshell Approach for Cardiac Arrest Caused by Hemorrhage of a Giant Mediastinal Ectopic Thyroid: A Case Report

**DOI:** 10.70352/scrj.cr.26-0494

**Published:** 2026-10-22

**Authors:** Minori Ono, Yui Watanabe, Takeo Togo, Yusuke Nakano, Ken Onodera, Hirotsugu Notsuda, Sakiko Kumata, Tetsuto Takeda, Takuya Ikushima, Tatsuaki Watanabe, Takaya Suzuki, Takashi Hirama, Hisashi Oishi, Yoshinori Okada

**Affiliations:** Department of Thoracic Surgery, Institute of Development, Aging and Cancer, Tohoku University, Sendai, Miyagi, Japan

**Keywords:** mediastinal ectopic thyroid, intratumoral hemorrhage, cardiac arrest, venoarterial extracorporeal membrane oxygenation, clamshell approach, emergency thoracotomy, mediastinal compression

## Abstract

**INTRODUCTION:**

Mediastinal ectopic thyroid tissue is rare and is usually asymptomatic. Intratumoral hemorrhage causing acute mediastinal compression and cardiac arrest is extremely uncommon. We report a case of cardiac arrest caused by hemorrhage of a giant mediastinal ectopic thyroid, successfully managed with venoarterial extracorporeal membrane oxygenation (VA-ECMO) and emergency surgical decompression by tumor removal.

**CASE PRESENTATION:**

A 71-year-old woman with a long-standing anterior mediastinal tumor was transferred to our hospital because of dyspnea and shock after a fall. Contrast-enhanced CT revealed a giant anterior mediastinal tumor occupying the left hemithorax, with active extravasation and marked mediastinal deviation. Transarterial embolization was performed; however, she developed cardiac arrest shortly after embolization, presumably due to rapid tumor enlargement and severe mediastinal compression caused by intratumoral hemorrhage. Cardiopulmonary resuscitation was immediately initiated, and VA-ECMO was established. Emergency tumor removal and hemostasis were performed through a 4th-intercostal clamshell incision. The tumor occupied almost the entire left thoracic cavity and was extensively adherent to the chest wall and mediastinum, with particularly firm adhesion to the left brachiocephalic vein. The tumor was removed with combined resection of the left brachiocephalic vein. Histopathological examination revealed benign ectopic thyroid tissue without malignant findings. The postoperative course was complicated by bleeding, infection, acute kidney injury, and prolonged impaired consciousness. Postoperative hypothyroidism was identified after pathological confirmation of ectopic thyroid tissue, and levothyroxine replacement was initiated. The patient was transferred to a rehabilitation hospital on POD 72 and is currently living at home 1 year after surgery.

**CONCLUSIONS:**

Giant mediastinal ectopic thyroid tissue can cause fatal circulatory collapse when complicated by intratumoral hemorrhage. Although the patient was in a critical condition, she was successfully rescued by VA-ECMO followed by emergency decompression through surgical tumor removal.

## Abbreviations

CPR
: cardiopulmonary resuscitation
ECMO
: extracorporeal membrane oxygenation
IVR
: interventional radiology
VA-ECMO
: venoarterial ECMO

## INTRODUCTION

Ectopic thyroid tissue is a rare developmental anomaly caused by abnormal migration of the thyroid primordium. Although it is most commonly located in the lingual region, ectopic thyroid tissue can also occur in the mediastinum.^1,2)^ Most cases are asymptomatic; however, large mediastinal lesions may cause symptoms by compressing adjacent organs.

Hemorrhage is an uncommon but potentially life-threatening complication of ectopic or orthotopic thyroid tissue. Previous reports have described massive bleeding or hematemesis from lingual ectopic thyroid tissue.^3,4)^ In addition, spontaneous hemorrhage of orthotopic thyroid lesions has been reported to cause airway compromise or acute respiratory failure through rapid cervical hematoma formation and laryngotracheal compression.^5)^ However, acute intratumoral hemorrhage of a mediastinal ectopic thyroid causing rapid mediastinal compression and cardiac arrest appears to be extremely rare.

ECMO has been used as a rescue or preventive strategy in patients with massive anterior mediastinal masses complicated by airway obstruction or cardiovascular collapse.^6,7)^ Nevertheless, reports describing the specific sequence of intratumoral hemorrhage of a mediastinal ectopic thyroid, cardiac arrest, VA-ECMO establishment during CPR, and emergency surgical decompression are limited. We report a rare case of cardiac arrest caused by hemorrhage of a giant mediastinal ectopic thyroid, successfully rescued by VA-ECMO and emergency decompression through surgical tumor removal.

## CASE PRESENTATION

A 71-year-old woman was transferred to our hospital because of dyspnea and shock after a fall. A mediastinal abnormality had been noted in another hospital more than 30 years earlier. Teratoma was suspected, and surgical resection had been recommended; however, the patient declined surgery and had been followed annually without apparent tumor growth or change in imaging characteristics. CT performed 6 months before the present episode showed a mass extending from the anterior mediastinum into the left thoracic cavity (**Fig. 1A**–**1C**).

**Fig. 1:**
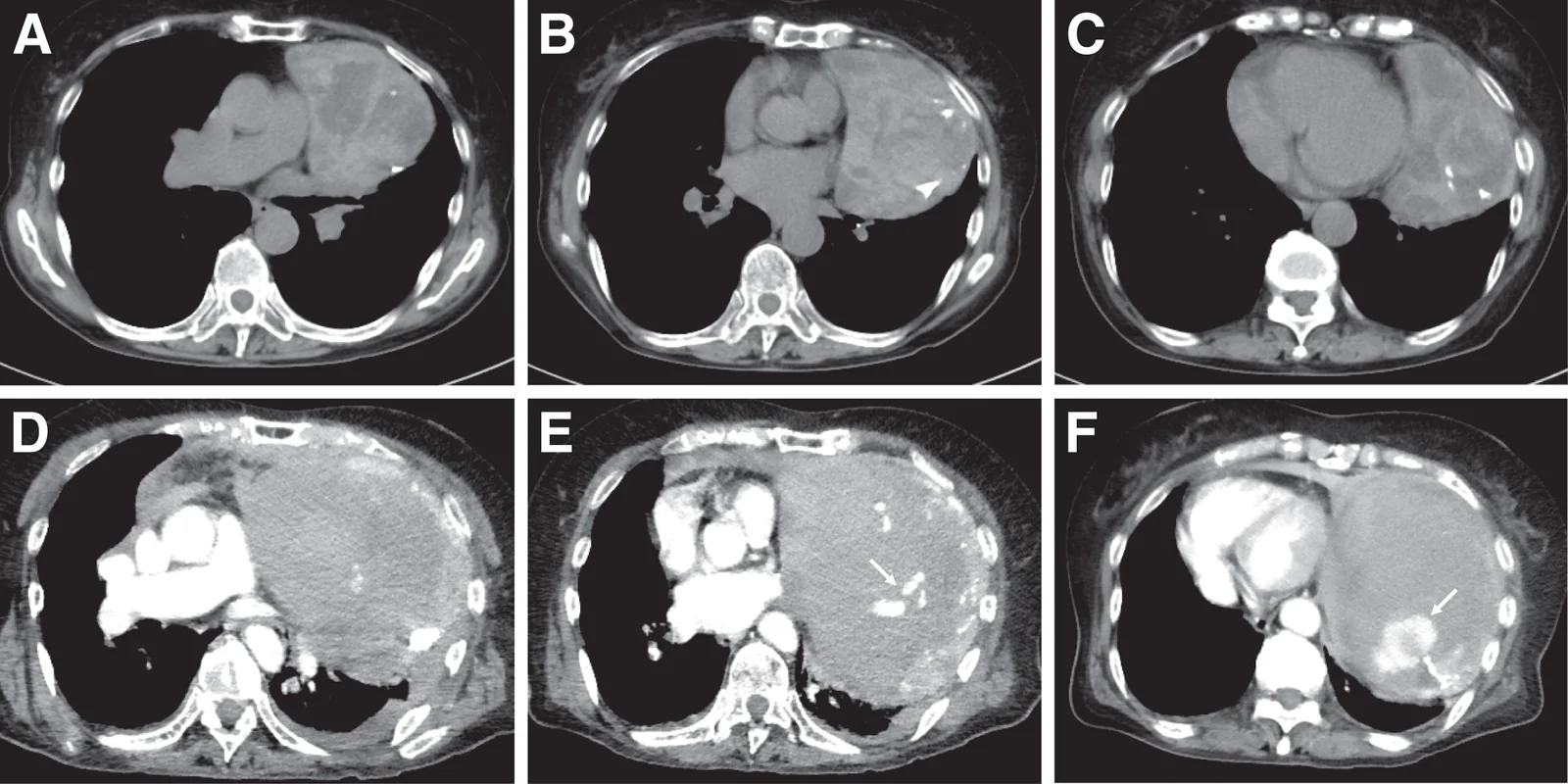
CT findings before and at presentation. CT performed 6 months before the acute event showed a giant anterior mediastinal tumor extending into the left thoracic cavity (**A**–**C**). Contrast-enhanced CT at presentation demonstrated marked tumor enlargement, contrast extravasation (arrows), intratumoral hemorrhage, and rightward mediastinal deviation (**D**–**F**).

Contrast-enhanced CT performed upon emergency admission to our hospital revealed a huge mediastinal tumor with active extravasation and rightward mediastinal deviation (**Fig. 1D**–**1F**). She was in shock, with anemia and elevated lactate levels. Thyroid function tests later confirmed biochemical hyperthyroidism; however, this was not recognized as clinically relevant during the initial emergency management.

Intratumoral hemorrhage from a giant anterior mediastinal tumor causing hemorrhagic shock was diagnosed. Because her blood pressure was temporarily maintained, active extravasation was evident, and IVR was more immediately available than surgical intervention, IVR was attempted. Angiography showed extravasation into the tumor, and transarterial embolization was performed. Extravasation was confirmed to have disappeared angiographically.

Shortly after embolization, the patient developed hemodynamic collapse and cardiac arrest, presumably because of rapid tumor enlargement and severe mediastinal compression caused by intratumoral hemorrhage and hematoma formation. CPR was immediately initiated, and VA-ECMO support was started 12 minutes after cardiac arrest. The patient was intubated, and VA-ECMO was established with venous drainage from the right femoral vein to the right atrium and arterial return via the left femoral artery. Because circulatory stability could not be maintained without decompression of the enlarging mediastinal mass, emergency surgical hemostasis and tumor removal were performed in the hybrid emergency operating room.

The chest was opened through a 4th-intercostal clamshell incision. A giant dark-colored tumor occupied almost the entire left thoracic cavity. It was widely adherent to the chest wall and mediastinum, with particularly firm adhesion to the left brachiocephalic vein. The tumor was removed with combined resection of the left brachiocephalic vein. An intraoperative autologous blood recovery system was not used because the surgery was performed as an emergency operation late at night in the hybrid operating room, detailed information regarding the underlying disease was unavailable, malignancy could not be completely excluded, and there was insufficient time to prepare the system. The operative time was 147 minutes, and total blood loss was 10521 mL. During surgery in the hybrid operating room, the patient required a massive transfusion consisting of 46 units of red blood cells, 34 units of fresh frozen plasma, and 20 units of platelet concentrate. The resected specimen consisted of multiple fragmented tumor components with extensive dark-red to black hematoma and hemorrhagic degeneration (**Fig. 2**).

**Fig. 2:**
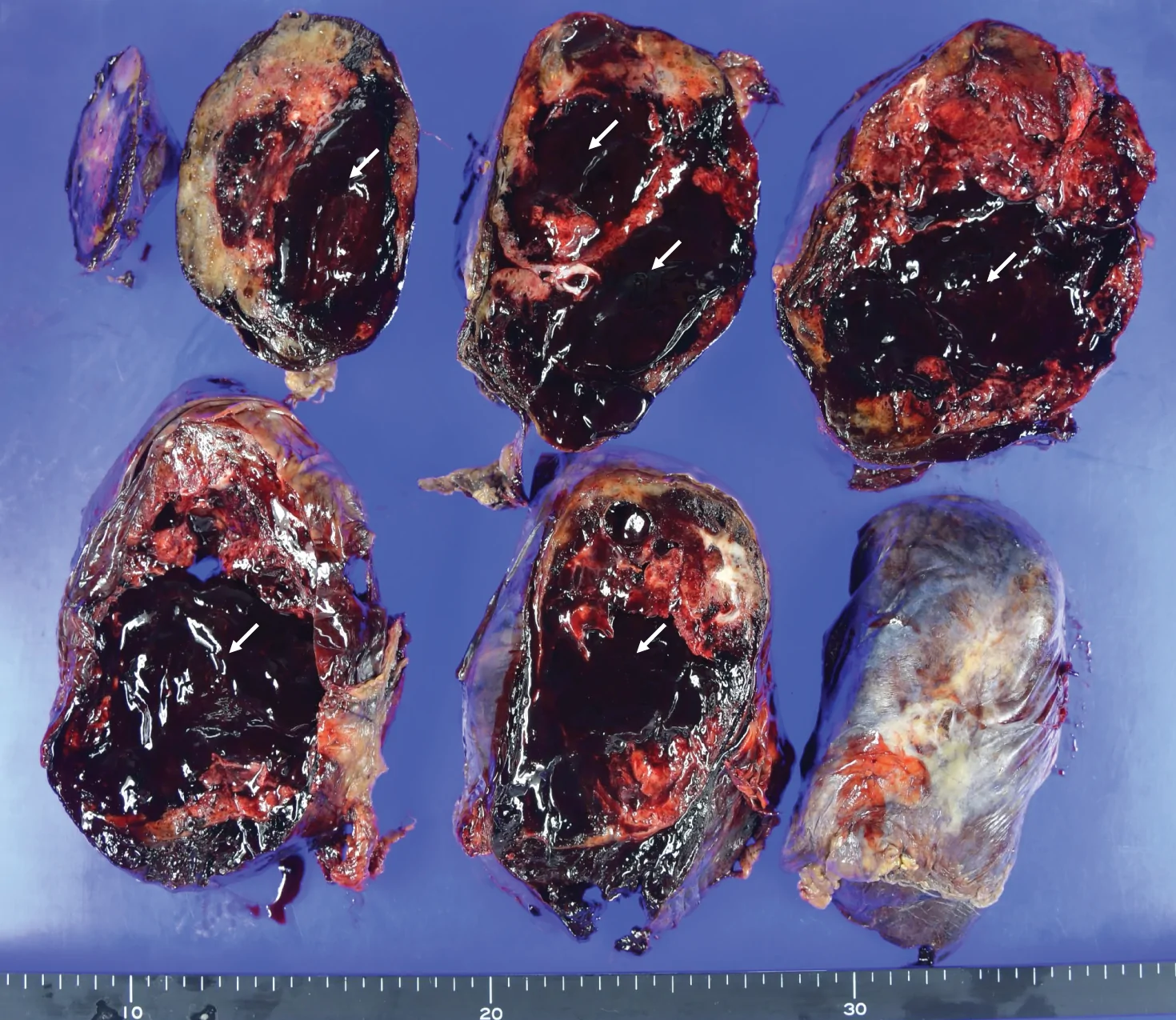
Macroscopic findings of the resected tumor. The resected specimen consisted of multiple fragmented tumor components. The cut surfaces showed extensive dark-red to black hematoma (arrows) and hemorrhagic degeneration, indicating massive intratumoral hemorrhage.

The patient was weaned from ECMO on POD 1. The postoperative course was complicated by persistent bleeding requiring reoperation for hematoma evacuation and hemostasis, surgical site infection, acute kidney injury requiring continuous hemodiafiltration, and prolonged impaired consciousness. Serial chest radiographs showed gradual improvement after emergency decompression and subsequent postoperative management (**Fig. 3**).

**Fig. 3:**
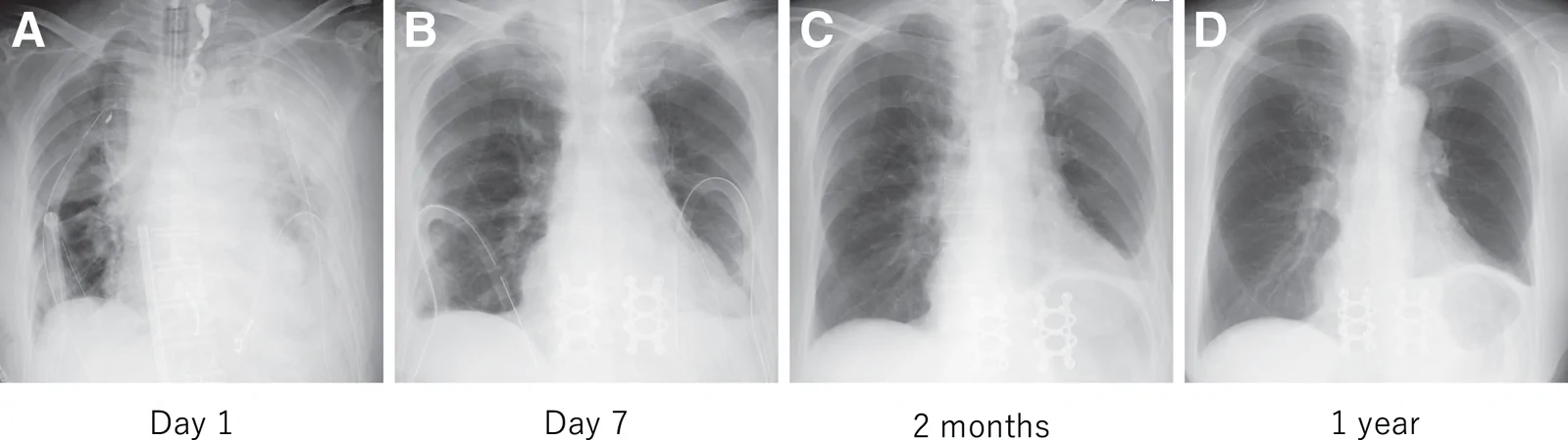
Postoperative chest radiographic course. Serial chest radiographs after emergency decompression showed gradual improvement. On POD 1, hematoma accumulation was observed (**A**) and required hematoma evacuation. By POD 7, bilateral lung expansion and aeration had improved (**B**), with further improvement at discharge (**C**). The patient showed a favorable course at 1 year after surgery (**D**).

Sedation was discontinued on POD 6, but impaired consciousness persisted. Head CT on POD 7 showed preserved gray–white matter differentiation, whereas electroencephalography showed generalized slowing. On POD 18, histopathological examination of the resected specimen revealed thyroid follicles of variable size with focal Sanderson polster–like changes. No features suggestive of papillary thyroid carcinoma or other malignancy were observed, confirming the diagnosis of benign ectopic thyroid tissue. At the same time, postoperative hypothyroidism was identified, and levothyroxine replacement was initiated on POD 19. Serial thyroid function tests obtained during the postoperative course up to 2 months after surgery are shown in **Fig. 4**. Although the prolonged impaired consciousness was probably multifactorial, thyroid hormone deficiency was considered a possible contributor to delayed neurological recovery. The patient subsequently recovered with rehabilitation and was transferred to a rehabilitation hospital on POD 72. One year after surgery, she was alert, able to communicate normally, and had no apparent cognitive impairment. She was able to eat a normal diet and was living at home with assistance, although residual lower limb weakness, neuropathic pain, and gait disturbance limited her ADL.

**Fig. 4:**
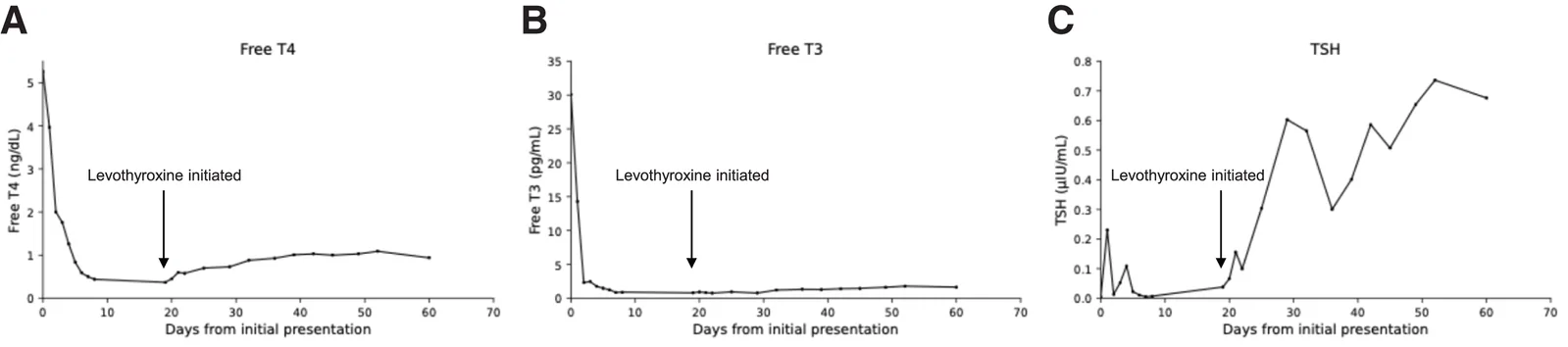
Postoperative changes in thyroid function during the acute phase. Serial changes in free T4 (**A**), free T3 (**B**), and TSH (**C**) from presentation to approximately 2 months after surgery. Levothyroxine was initiated on approximately POD 19. T3, triiodothyronine; T4, thyroxine; TSH, thyroid-stimulating hormone

## DISCUSSION

This case suggests that intratumoral hemorrhage of a giant mediastinal ectopic thyroid may cause severe circulatory collapse through rapid mediastinal compression. It also illustrates that surgical decompression may need to be considered when the mass effect of a hemorrhagic mediastinal tumor remains life-threatening, even after angiographic hemostasis.

The clinical management of mediastinal ectopic thyroid tissue remains challenging because of its rarity and variable presentation. Surgical treatment is generally considered for symptomatic lesions, suspected malignancy, large lesions with compressive effects, or hemorrhagic complications.^1,2)^ However, there is no established consensus regarding the optimal timing of surgery for asymptomatic large mediastinal lesions. In the present case, the tumor had been observed for years without apparent change, but minor trauma was followed by intratumoral hemorrhage, rapid enlargement, and severe mediastinal compression. Although this single case cannot determine surgical indications, it suggests that even apparently stable giant mediastinal ectopic thyroid tissue may become life-threatening if hemorrhage occurs.

Previous reports of hemorrhagic ectopic thyroid tissue have mainly involved cervical or lingual lesions, in which bleeding can cause airway symptoms, hematemesis, or shock.^3,4)^ Spontaneous hemorrhage of an orthotopic thyroid lesion can also cause acute airway compromise or respiratory failure.^5)^ In contrast, the present lesion was located in the mediastinum and extended into the left thoracic cavity; therefore, hemorrhage resulted primarily in acute mediastinal compression and cardiac arrest. This anatomical and physiological sequence distinguishes the present case from previously reported hemorrhagic thyroid lesions.

IVR was initially selected because the patient’s blood pressure was temporarily maintained, active extravasation was evident on contrast-enhanced CT, and IVR was more immediately available than surgical intervention. Although angiographic embolization eliminated the visible extravasation, hemodynamic collapse occurred soon afterward. This suggests that the mass effect of the enlarged hemorrhagic tumor and intratumoral hematoma, rather than active bleeding alone, was critical. Therefore, hemostasis alone was insufficient, and urgent surgical decompression was required. In bleeding mediastinal tumors, treatment strategies should address both hemorrhage control and relief of mediastinal compression.

The initial shock was mainly attributable to intratumoral hemorrhage, as evidenced by anemia, elevated lactate levels, and active extravasation on contrast-enhanced CT. However, hemodynamic collapse and cardiac arrest occurred shortly after angiographic hemostasis had been achieved, suggesting that progressive tumor enlargement due to intratumoral hematoma also contributed to the subsequent circulatory collapse.

ECMO has been reported as a useful support strategy for patients with massive anterior mediastinal masses who develop airway obstruction or cardiovascular collapse during sedation, biopsy, anesthesia induction, or definitive treatment.^6,7)^ In the present case, VA-ECMO was established during CPR and enabled immediate surgery. The absence of no-flow time, the short low-flow time, and prompt decompression were likely essential for survival.

The surgical approach was also important. Median sternotomy is standard for anterior mediastinal tumors, but this lesion was giant, occupied almost the entire left hemithorax, and had an unclear relationship with the great vessels. A clamshell approach provides wide exposure of the thoracic cavity and mediastinum and has been reported to provide superior access to intrathoracic structures in emergency thoracotomy.^8)^ Although it may be associated with wound complications and postoperative morbidity,^9)^ it remains valuable when rapid and wide exposure is required for life-threatening mediastinal compression or bleeding. Although a left anterolateral thoracotomy or unilateral hemi-clamshell approach might have been considered because the tumor was located predominantly in the left hemithorax, the patient was hemodynamically unstable after cardiac arrest and the tumor was extremely large. Therefore, we selected a clamshell approach to obtain a bilateral surgical field and sufficient working space for rapid decompression, hemostasis, and vascular control if required.

Finally, this case highlights the importance of postoperative endocrine evaluation. The patient had biochemical hyperthyroidism before surgery, but this was not recognized during the initial emergency phase. After resection of functional ectopic thyroid tissue, postoperative hypothyroidism was identified. Prolonged impaired consciousness was initially attributed mainly to hypoxic brain injury; however, the clinical course was probably multifactorial, including cardiac arrest, critical illness, sedation, renal dysfunction, infection, and thyroid hormone deficiency. Therefore, levothyroxine replacement was initiated after pathological confirmation of ectopic thyroid tissue and the detection of postoperative hypothyroidism. As shown in **Fig. 4**, postoperative thyroid function showed a complex course, and the delayed neurological recovery was likely multifactorial rather than attributable solely to thyroid hormone deficiency.

This single emergency case cannot define an optimal management strategy. We do not intend to suggest that all long-standing benign mediastinal tumors should be surgically resected solely because of the possibility of hemorrhage, nor that tumor resection should always be prioritized over IVR in similar cases. Rather, the key clinical message is that treatment strategies for hemorrhagic mediastinal tumors should be individualized and should address both hemorrhage control and relief of mediastinal compression. When hemodynamic collapse persists or progresses despite hemostasis, VA-ECMO–supported emergency surgical decompression may be lifesaving.

## CONCLUSIONS

This case highlights that hemostasis alone may be insufficient in hemorrhagic mediastinal tumors when progressive intratumoral hematoma causes life-threatening mediastinal compression. In patients with persistent or worsening hemodynamic collapse despite angiographic hemostasis, timely emergency surgical decompression should be considered as a lifesaving strategy.

## DECLARATIONS

### Use of artificial intelligence (AI) tools

The authors used ChatGPT, a large language model developed by OpenAI, for English-language editing and manuscript formatting. Perplexity was used to support preliminary literature searches.

The authors reviewed and verified all content, references, and interpretations, and take full responsibility for the final manuscript.

### Availability of data and materials

The data supporting the conclusions of this article are included within the article.
